# The exact analytical solution of the dual-phase-lag two-temperature bioheat transfer of a skin tissue subjected to constant heat flux

**DOI:** 10.1038/s41598-020-73086-0

**Published:** 2020-09-29

**Authors:** Hamdy M. Youssef, Najat A. Alghamdi

**Affiliations:** 1 Mechanical Engineering Department, College of Engineering and Islamic Architecture, Umm Al-Qura University, Mecca, Saudi Arabia; 2 Department of Mathematics, Faculty of Applied Science, Umm Al-Qura University, Mecca, Saudi Arabia

**Keywords:** Biological techniques, Biophysics, Computational biology and bioinformatics

## Abstract

This work is dealing with the temperature reaction and response of skin tissue due to constant surface heat flux. The exact analytical solution has been obtained for the two-temperature dual-phase-lag (TTDPL) of bioheat transfer. We assumed that the skin tissue is subjected to a constant heat flux on the bounding plane of the skin surface. The separation of variables for the governing equations as a finite domain is employed. The transition temperature responses have been obtained and discussed. The results represent that the dual-phase-lag time parameter, heat flux value, and two-temperature parameter have significant effects on the dynamical and conductive temperature increment of the skin tissue. The Two-temperature dual-phase-lag (TTDPL) bioheat transfer model is a successful model to describe the behavior of the thermal wave through the skin tissue.

## Introduction

The thermal transfer through a living skin tissue is extremely essential to a wide range of therapies and their applications^1^. Improvements in the microwave, laser, and other technologies also extended bioheat transfer work. Several researchers comment on biothermal transmission analyzes in biological living tissues, including Pennes^2^. He optimized the parabolic model for biological tissues for the first bioheat transfer. Also, the Pennes biological transmission method is used to model the nature and consistency of thermal behavior in biological tissues and living bodies. External similarities to such unpredictable results that display non-Fourier or hyperbolic conduction behavior. Cattaneo^3^ and Vernotte^4^ provided different modulations of Fourier's law of heat conduction as a linear extension form of the Fourier law to describe the hyperbolic equation type. They suggested a model of thermal waves measure the effect of microwave and thermal flux activity. Different forms of work have been undertaken to address various skin tissue conditions without damaging the balanced tissue surrounding them. Xu et al.^5^ solved analytical the Pennes bioheat transfer equation (PBTE), reviewed skin bioheat transfer, skin structure, thermal damage, skin biomechanics, and bio-thermomechanics. The non-Fourier thermomechanical activity of skin tissues under various surface thermal loading limits was analyzed in DPL, hyperbolic and parabolic models of biomass transport, Xu and al. studied and noticed substantial variations between Pennes, thermal wave and DPL anticipations models^6^. Also, Rossmann et al.^7^ reviewed the temperature dependence of the dielectric properties, thermal properties, and perfusion of biological tissues at hyper-thermic and ablation temperatures. Poor et al.^8^ focused on the temperature disturbance of skin tissue due to time-dependent surface heating. Tzou's model has been extended by the dual-phase-lag principle, with the delayed activity in a high rate of response taken into consideration. Although the process is lagging, the small-scale response is caught in time^9,10^. Tzou^11^ introduced a phase-lag for a temperature gradient. Askarizadeh et al.^12^ utilized the dual-phase-lag (DPL) model in treating the transient heat transfer problems in skin tissue. Dutta and Kundu established a two-dimensional thermal model of malignant tissues, focused on the bi-dimensional local thermal non-balance therapy model for bio-heat^13^.

Liu et al. first address the overall bioheat transmission paradigm in living tissue and analyze^14^. Liu et al. are tested during the hyperthermia procedure in the two-stage model for heat transfer problems in the biological tissue^15,16^. The Liu and Chen thermal conductor model (DPL) used the non-Fourier thermal activity for the diagnosis of hyperthermia to describe the thermal transportation occurring in biological tissue^17^. In terms of the properties of blood and tissue and the interphase charge of the heat transfer parameter and perfusion rate, Zhang articulated phase lag or relaxation periods^18^. He observed that the lap periods are very similar together for living tissues. Dutta and Kundu performed a thermal wave propagation study for a constant and variable flux of heat on a skin tissue surface in biological tissue, connected with the diagnosis of hyperthermia.^19^.

A finite element approach to the temperature distribution of deep skin tissues in the human limb was suggested by Agrawal et al.^20^. Kumar et al. also researched the thermal behavior, using a dual-phase-lag (DPL) model of time-fractional^21^. To address the effect of injecting nanoparticles in irregularly-shaped liver tumors on cancer cells, Shao et al. have developed an RFA model^22^. A dual-phase-lag (DPL) bioheat transfer model analysis approach was used by Poor et al. on the skin tissue as the finite domain of continuous, cosine, and pulse heat flow conditions on the surface of the skin^8^.

Liu and Xu developed a closed-form analytical approach for the Pennes bioheat equation solution for changes in skin tissue temperature induced by sinusoidal heat flow^23^. Shih et al. have overcome the consequence of a semi-infinite biological skin tissue temperature reaction study undergoing sine flux^24^. Regarding periodic constant and pulsed train heat flux boundary conditions, Ahmadika et al. shall have the analytical solution for the bioheat (parabolize) and hyperbolized bioheat transfer models of the Penne^25^. The statistical model was used by Horng et al. to explain the effects of pulsatile blood flow in the thermal disruption^26^. He found out that the influence on the thermal area on tumor and the slight variation only on a blood continuum range from standard to parabolic, velocity profiles on the blood flow. Shih et al. investigated the coupled effects of the pulsatile blood flow and thermal relaxation time in living tissues during thermal treatments^27^. Youssef and Alghamdi^28^ have solved a one-dimensional application of thermoelastic dual-phase-lag skin tissue under specific thermal loading. Kundu and Dewanjee^29^ introduced a new method for non-Fourier thermal response in a single layer skin tissue.

For one calming period in deformable structures, Youssef updated Chen and Gurtin's principle of thermal actions. Two specific temperatures are the thermodynamic temperature and the conductive temperature in this device^30,31^. The disparity in the meaning of these two temperature forms is commensurate with the importance of the material's thermal supply. Youssef with El-Bary and Alghamdi applied the two-temperature heat conduction in many applications^32–37^.

In the present paper, a mathematical model of bioheat skin tissue will be constructed in the context of a two-temperature dual-phase-lag heat conduction model. The surface of the skin tissue is subjected to a constant heat flux. The separation of variables as a finite domain will be applied to obtain the exact analytical solution. The effects of the dual-phase-lag time parameter, value of the heat flux, and two-temperature parameter will be studied and discussed.

### Bioheat transfer models for biological tissues

The bioheat transmitting model was developed to measure the time-dependent temperature change as a heat reaction due to the usage of either heat source or thermal heating. The first model of biological tissues was found out by Pennes based on Fourier's law of heat conduction as follows^28,36,37^:

1 $$ K\nabla^{2} T = \rho C\frac{\partial T}{{\partial t}} - \rho_{b} w_{b} C_{b} \left( {T_{b} - T} \right) - \left( {Q_{met} + Q_{ext} } \right), $$

where $\rho ,\,C$ and $K$ are the density, specific heat, and thermal conductivity of the tissue, respectively.$C_{b} ,\,w_{b} \,$, $\rho_{b}$ and $T_{b}$ denote the specific heat of the blood, blood perfusion rate, blood density, and blood temperature, respectively. *T* is the absolute temperature function. $Q_{met}$ denotes the metabolic heat generated by the chemical reaction inside the tissue, and it is constant, and the external heat source is given by $Q_{ext} = Q_{ext} \left( t \right)$. $\nabla^{2}$ is the well-known Laplace operator.

### The modified Fourier's law of heat conduction

Ventott and Cattaneo modified the classical Fourier thermal drive law by postulating the concept of finite thermal wave propagation speed and taking the following hyperbolic form of the thermal wave^28,36,37^:

2 $$ K\nabla^{2} T = \rho C\left( {1 + \tau_{q} \frac{\partial }{\partial t}} \right)\frac{\partial T}{{\partial t}} + w_{b} C_{b} \rho_{b} \left( {1 + \tau_{q} \frac{\partial }{\partial t}} \right)\left( {T - T_{b} } \right) - \left( {1 + \tau_{q} \frac{\partial }{\partial t}} \right)\left( {Q_{met} + Q_{ext} } \right), $$

where $\tau_{q} = \frac{\alpha }{{c_{0}^{2} }} > 0$ is a material property and is called the relaxation time parameter, and $\alpha$ is the thermal diffusivity while $c_{0}$ is the speed of the thermal wave inside the medium.

### Dual-phase-lag model (DPL) of bioheat transfer

The dual-phase-lag (DPL) model based on the dual reaction between the gradient of the temperature $\nabla T$ and the heat flux $q$ which modified the well-known classical Fourier's law of heat conduction, thus, we have the following heat conduction equation^28,36,37^:

3 $$ K\left( {1 + \tau_{T} \frac{\partial }{\partial t}} \right)\nabla^{2} T = \rho C\left( {1 + \tau_{q} \frac{\partial }{\partial t}} \right)\frac{\partial T}{{\partial t}} + w_{b} C_{b} \rho_{b} \left( {1 + \tau_{q} \frac{\partial }{\partial t}} \right)\left( {T - T_{b} } \right) - \left( {1 + \tau_{q} \frac{\partial }{\partial t}} \right)\left( {Q_{met} + Q_{ext} } \right) $$

where $\tau_{T} \ge 0$ is the second parameter of the relaxation time, which is the phase-lag of the temperature gradient passing through the medium.

### Two-temperature dual-phase-lag (TTDPL) bioheat transfer model on skin tissues

According to Youssef's model of heat conduction formulation, we have two equations of heat conduction on the biological tissues as following^36,37^ :

4 $$ K\left( {1 + \tau_{T} \frac{\partial }{\partial t}} \right)\nabla^{2} T_{C} = \rho C\left( {1 + \tau_{q} \frac{\partial }{\partial t}} \right)\frac{{\partial T_{D} }}{\partial t} + w_{b} C_{b} \rho_{b} \left( {1 + \tau_{q} \frac{\partial }{\partial t}} \right)T_{D} - \left( {1 + \tau_{q} \frac{\partial }{\partial t}} \right)\left( {Q_{met} + Q_{ext} } \right) $$

and

5 $$ T_{D} = T_{C} - \beta \nabla^{2} T_{C} $$

where $\beta$ is a non-negative constant, which is called the two-temperature parameter^36,37^. $T_{D}$ and $T_{C}$ are the dynamical temperature and the conductive temperature, respectively. The value $\beta = 0$ represents the one-temperature model, then, we obtain $T_{C} = T_{D}$.

We consider the function of dynamical temperature increment takes the form

6 $$ \theta = T_{D} - T_{b} $$

and the function of conductive temperature increment takes the form

7 $$ \varphi = T_{C} - T_{b} $$

Hence, we have

8 $$ K\left( {1 + \tau_{T} \frac{\partial }{\partial t}} \right)\nabla^{2} \varphi = \rho C\left( {1 + \tau_{q} \frac{\partial }{\partial t}} \right)\frac{\partial \theta }{{\partial t}} + w_{b} C_{b} \rho_{b} \left( {1 + \tau_{q} \frac{\partial }{\partial t}} \right)\theta - \left( {1 + \tau_{q} \frac{\partial }{\partial t}} \right)\left( {Q_{met} + Q_{ext} } \right) $$

and

9 $$ \theta = \varphi - \beta \,\nabla^{2} \varphi $$

## Method

**Figure 1:**
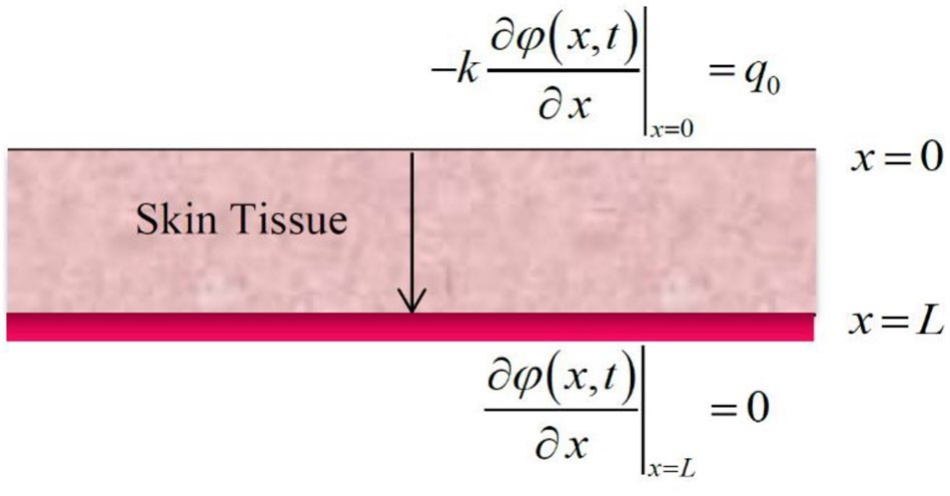
The skin tissue affected by constant heat flux on the surface.

We consider the region $0 \le x \le L$ is filled with skin tissue as in Fig. 1 and obeys the two-temperature dual-phase-lag (TTDPL) as in Eqs. (8) and (9).

The medium is considered to be quiescent initially and without any external heat source $\left( {Q_{ext} = 0} \right)$ while $Q_{met}$ is constant. The skin is subjected to a constant heat flux $q_{0}$ on its bounding surface $x = 0$, while the bounding surface $x = L$ has no heat flux (see Fig. 1).

Hence, the heat conduction equations take the forms:

10 $$ \left[ {\beta \rho C\tau_{q} \frac{{\partial^{2} }}{{\partial t^{2} }} + \left( {K\tau_{T} + \beta \rho C + \beta \tau_{q} w_{b} C_{b} \rho_{b} } \right)\frac{\partial }{\partial t} + \left( {\beta w_{b} C_{b} \rho_{b} + K} \right)} \right]\frac{{\partial^{2} \varphi }}{{\partial x^{2} }} = \left( {\tau_{q} \rho C\frac{{\partial^{2} }}{{\partial t^{2} }} + \left( {\rho C + \tau_{q} w_{b} C_{b} \rho_{b} } \right)\frac{\partial }{\partial t} + w_{b} C_{b} \rho_{b} } \right)\varphi - Q_{met} $$

and

11 $$ \theta = \varphi - \beta \frac{{\partial^{2} \varphi }}{{\partial x^{2} }} $$

The initial conditions are:

12 $$ \left. {\varphi \left( {x,t} \right)} \right|_{t = 0} \left. { = \theta \left( {x,t} \right)} \right|_{t = 0} = \left. {\frac{{\partial \varphi \left( {x,t} \right)}}{\partial t}} \right|_{t = 0} = \left. {\frac{{\partial \theta \left( {x,t} \right)}}{\partial t}} \right|_{t = 0} = 0 $$

The boundary conditions are:

13 $$ \left. { - K\frac{{\partial \varphi \left( {x,t} \right)}}{\partial \,x}} \right|_{x = 0} = q_{0} \quad \left. {\frac{{\partial \varphi \left( {x,t} \right)}}{\partial \,x}} \right|_{x = L} = 0 $$

The boundary value problem (10)–(13) consists of non-homogeneous partial differential equations with non-homogeneous boundary conditions on the surface of the skin tissue. Thus, the equations must be formulated in terms of a steady part and a transient part as follows^38,39^:

14 $$ \varphi \left( {x,t} \right) = \phi_{1} \left( {x,t} \right) + \phi_{2} \left( x \right) $$

Hence, we obtain the transient part in the form

15 $$ \left[ {\beta \rho C\tau_{q} \frac{{\partial^{2} }}{{\partial t^{2} }} + \left( {K\tau_{T} + \beta \rho C\, + \beta \tau_{q} w_{b} C_{b} \rho_{b} } \right)\frac{\partial }{\partial t} + \left( {\beta w_{b} C_{b} \rho_{b} + K} \right)} \right]\frac{{\partial^{2} \varphi_{1} \left( {x,t} \right)}}{{\partial x^{2} }} - \left( {\tau_{q} \rho C\frac{{\partial^{2} }}{{\partial t^{2} }} + \left( {\rho C + \tau_{q} w_{b} C_{b} \rho_{b} } \right)\frac{\partial }{\partial t} + w_{b} C_{b} \rho_{b} } \right)\varphi_{1} \left( {x,t} \right) = 0 $$

and the steady-state part in the form

16 $$ \left( {\frac{{d^{2} }}{{dx^{2} }} - \lambda^{2} } \right)\phi_{2} \left( x \right) = - \gamma \psi $$

where $$\lambda^{2} = \frac{{w_{b} C_{b} \rho_{b} }}{{\left( {\beta w_{b} C_{b} \rho_{b} + K} \right)}} > 0,\quad \gamma = \frac{1}{{\left( {\beta w_{b} C_{b} \rho_{b} + K} \right)}} > 0$$, and $$\psi = Q_{met}$$.

The boundary conditions for the steady-state equation are

17 $$ - K\left. {\frac{{d\phi_{2} \left( x \right)}}{dx}} \right|_{x = 0} = q_{0} ,\quad - K\left. {\frac{{d\phi_{2} \left( x \right)}}{dx}} \right|_{x = L} = 0 $$

Then, regarding the boundary conditions on (13), the solution of the Eq. (10) is in the form (See “Appendix”):

18 $$ \phi_{2} \left( x \right) = \frac{{q_{0} \cosh \lambda \left( {L - x} \right)}}{\lambda K\sinh \lambda L} + \frac{\gamma }{{\lambda^{2} }}\psi $$

The initial and boundary conditions of the transient Eq. (12) are:

19 $$ \left. {\phi_{1} \left( {x,t} \right)} \right|_{t = 0} = - \phi_{2} \left( x \right),\quad \left. {\frac{{\partial \phi_{1} \left( {x,t} \right)}}{\partial t}} \right|_{t = 0} = 0 $$

and

20 $$ \left. {\frac{{\partial \phi_{1} \left( {x,t} \right)}}{\partial x}} \right|_{x = 0} = 0,\quad \left. {\frac{{\partial \phi_{1} \left( {x,t} \right)}}{\partial x}} \right|_{x = L} = 0 $$

To solve the Eq. (15) we expand the function $\phi_{1} \left( {x,t} \right)$ in the following Fourier series expansion^38,39^

21 $$ \phi_{1} \left( {x,t} \right) = \sum\limits_{n = 0}^{\infty } {\vartheta_{n} \left( t \right)\cos \left( {\frac{n\pi }{L}x} \right)} $$

which satisfies the boundary conditions (20).

Substitute from Eqs. (21) into (15), we get

22 $$ \left[ {\frac{{\partial^{2} }}{{\partial t^{2} }} + A_{1n} \frac{\partial }{\partial t} + A_{2n} } \right]\vartheta_{n} \left( t \right) = 0,\quad n = 0,1,2,3 \ldots $$

where $$A_{1n} = \frac{{\omega_{n}^{2} \left( {\eta \tau_{T} + \beta + \beta \tau_{q} \varepsilon } \right) + \left( {1 + \tau_{q} \varepsilon } \right)}}{{\tau_{q} \left( {\beta \omega_{n}^{2} + 1} \right)}},\;\;A_{2n} = \frac{{\omega_{n}^{2} \left( {\beta \varepsilon + \eta } \right) + \varepsilon }}{{\tau_{q} \left( {\beta \omega_{n}^{2} + 1} \right)}},\;\;\varepsilon = \frac{{w_{b} C_{b} \rho_{b} }}{\rho C},\;\;\eta = \frac{K}{\rho C}$$ and $$\omega_{n} = \frac{n\pi }{L}$$.

The general solutions to (22) take the following forms^38,39^:

23 $$ \vartheta_{n} \left( t \right) = a_{n} f_{1} \left( {k_{1n} ,t} \right) + b_{n} f_{2} \left( {k_{2n} ,t} \right),\quad n = 0,1,2, \ldots $$

which give

24 $$ \vartheta_{1} \left( {x,t} \right) = \sum\limits_{n = 0}^{\infty } {\left[ {a_{n} f_{1} \left( {k_{1n} ,t} \right) + b_{n} f_{2} \left( {k_{2n} ,t} \right)} \right]} \cos \left( {\omega_{n} x} \right) $$

where $k_{1n} ,k_{2n}$ is the solution of the following characteristic equation

25 $$ k^{2} + A_{1n} k + A_{2n} = 0 $$

The roots of the characteristic Eq. (25) are in the forms:

26 $$ k_{1n} = \frac{{ - A_{1n} + \sqrt {\Delta_{n} } }}{2},\quad k_{2n} = \frac{{ - A_{1n} - \sqrt {\Delta_{n} } }}{2} $$

where $$\,\Delta_{n} = A_{1n}^{2} - 4A_{2n}$$.

To apply the initial conditions (19), we have to expand $\phi_{2} \left( x \right)$ in Fourier series expansion to be in the form:

27 $$ \phi_{2} \left( x \right) = \frac{{q_{0} }}{{2LK\lambda^{2} }} + \frac{\gamma \psi }{{2\lambda^{2} }} + \frac{{q_{0} }}{KL}\sum\limits_{n = 1}^{\infty } {\frac{1}{{\left( {\lambda^{2} + \omega_{n}^{2} } \right)}}} \cos \left( {\omega_{n} x} \right) $$

Hence, the initial conditions (19) give the following system of algebraic equations:

28 $$ \left[ {a_{0} f_{1} \left( {k_{10} ,0} \right) + b_{0} f_{2} \left( {k_{20} ,0} \right)} \right]\cos \left( {\omega_{0} x} \right) + \sum\limits_{n = 1}^{\infty } {\left[ {a_{n} f_{1} \left( {k_{1n} ,0} \right) + b_{n} f_{2} \left( {k_{2n} ,0} \right)} \right]} \cos \left( {\omega_{n} x} \right) = - \phi_{2} \left( x \right) $$

and

29 $$ \left[ {a_{0} \frac{{\partial f_{1} \left( {k_{10} ,0} \right)}}{\partial t} + b_{0} \frac{{\partial f_{2} \left( {k_{20} ,0} \right)}}{\partial t}} \right]\cos \left( {\omega_{0} x} \right) + \sum\limits_{n = 1}^{\infty } {\left[ {a_{n} \frac{{\partial f_{1} \left( {k_{1n} ,0} \right)}}{\partial t} + b_{n} \frac{{\partial f_{2} \left( {k_{2n} ,0} \right)}}{\partial t}} \right]} \cos \left( {\omega_{n} x} \right) = 0 $$

For the case of $\Delta_{n} > 0$

30 $$ f_{1} \left( {k_{1n} ,t} \right) = {\text{e}}^{{k_{1n} \,t}} \;\; {\text{and}}\;\;f_{2} \left( {k_{2n} ,t} \right) = {\text{e}}^{{k_{2n} \,t}} $$

Thus, when $t = 0$ we have

$$ f_{1} \left( {k_{1n} ,0} \right) = f_{2} \left( {k_{2n} ,0} \right) = 1,\quad \frac{{\partial f_{1} \left( {k_{1n} ,0} \right)}}{\partial t} = k_{1n} ,\quad \frac{{\partial f_{2} \left( {k_{2n} ,0} \right)}}{\partial t} = k_{2n} ,\quad n = 0,1,2,3 \ldots $$

Then, Eqs. (28) and (29) introduce the following system

31 $$ \left[ {a_{0} + b_{0} } \right]\cos \left( {\omega_{0} x} \right) + \sum\limits_{n = 1}^{\infty } {\left[ {a_{n} + b_{n} } \right]} \cos \left( {\omega_{n} x} \right) = - \frac{{q_{0} }}{{2LK\lambda^{2} }} - \frac{\gamma \psi }{{2\lambda^{2} }} - \frac{{q_{0} }}{KL}\sum\limits_{n = 1}^{\infty } {\frac{1}{{\left( {\lambda^{2} + \omega_{n}^{2} } \right)}}} \cos \left( {\omega_{n} x} \right) $$

and

32 $$ \left[ {k_{10} a_{0} + k_{20} b_{0} } \right]\cos \left( {\omega_{0} x} \right) + \sum\limits_{n = 1}^{\infty } {\left[ {k_{1n} a_{n} + k_{2n} b_{n} } \right]} \cos \left( {\omega_{n} x} \right) = 0 $$

when $n = 0$ we have

$$ \omega_{0} = 0,\;\cos \left( {\omega_{0} x} \right) = 1,\;A_{10} = \frac{{\varepsilon \tau_{q} + 1}}{{\tau_{q} }},\,\,\,A_{20} = \frac{\varepsilon }{{\tau_{q} }},\,\Delta_{0} = \left( {\frac{{\varepsilon \tau_{q} - 1}}{{\tau_{q} }}} \right)^{2} ,\,k_{10} = \frac{ - 1}{{\tau_{q} }},\;k_{20} = - \varepsilon . $$

Hence, the system in Eqs. (31) and (32) will be reduced to the following system

33 $$ a_{0} + b_{0} + \sum\limits_{n = 1}^{\infty } {\left[ {a_{n} + b_{n} } \right]} \cos \left( {\omega_{n} x} \right) = - \frac{{q_{0} }}{{2LK\lambda^{2} }} - \frac{\gamma \psi }{{2\lambda^{2} }} - \frac{{q_{0} }}{LK}\sum\limits_{n = 1}^{\infty } {\frac{1}{{\left( {\lambda^{2} + \omega_{n}^{2} } \right)}}} \cos \left( {\omega_{n} x} \right) $$

and

34 $$ - \frac{{a_{0} }}{{\tau_{q} }} - \varepsilon b_{0} + \sum\limits_{n = 1}^{\infty } {\left[ {k_{1n} a_{n} + k_{2n} b_{n} } \right]} \cos \left( {\omega_{n} x} \right) = 0 $$

The equations in (33) and (34) lead to the following two systems of algebraic equations

35 $$ a_{0} + b_{0} = - \left( {\frac{{q_{0} }}{{2LK\lambda^{2} }} + \frac{\gamma \psi }{{2\lambda^{2} }}} \right),\quad a_{0} + \tau_{q} \varepsilon b_{0} = 0 $$

and

36 $$ a_{n} + b_{n} = \frac{{ - q_{0} }}{{LK\left( {\lambda^{2} + \omega_{n}^{2} } \right)}},\quad k_{1n} a_{n} + k_{2n} b_{n} = 0,\quad n = 1,2,3, \ldots $$

By solving the above two systems, we get

37 $$ a_{0} = \left( {\frac{{\varepsilon \tau_{q} }}{{1 - \varepsilon \tau_{q} }}} \right)\left( {\frac{{q_{0} }}{{2LK\lambda^{2} }} + \frac{\gamma \psi }{{2\lambda^{2} }}} \right),\quad b_{0} = \left( {\frac{ - 1}{{1 - \varepsilon \tau_{q} }}} \right)\left( {\frac{{q_{0} }}{{2LK\lambda^{2} }} + \frac{\gamma \psi }{{2\lambda^{2} }}} \right) $$

and

38 $$ a_{n} = \left( {\frac{{k_{2n} }}{{k_{1n} - k_{2n} }}} \right)\frac{{q_{0} }}{{LK\left( {\lambda^{2} + \omega_{n}^{2} } \right)}},\quad b_{n} = \left( {\frac{{ - k_{1n} }}{{k_{1n} - k_{2n} }}} \right)\frac{{q_{0} }}{{LK\left( {\lambda^{2} + \omega_{n}^{2} } \right)}},\quad n = 1,2,3 \ldots $$

Now, the final solution of the heat conduction temperature increment, in this case, is in the form

39 $$ \varphi \left( {x,t} \right) = \frac{{q_{0} }}{{2LK\lambda^{2} }} + \frac{\gamma \psi }{{2\lambda^{2} }} + \frac{{q_{0} }}{KL}\sum\limits_{n = 1}^{\infty } {\frac{1}{{\left( {\lambda^{2} + \omega_{n}^{2} } \right)}}} \cos \left( {\omega_{n} x} \right) + \left( {\frac{1}{{1 - \varepsilon \tau_{q} }}} \right)\left( {\frac{{q_{0} }}{{2LK\lambda^{2} }} + \frac{\gamma \psi }{{2\lambda^{2} }}} \right)\left[ {\varepsilon \tau_{q} {\text{e}}^{{\frac{ - t}{{\tau_{q} }}}} - {\text{e}}^{ - \varepsilon t} } \right] + \frac{{q_{0} }}{KL}\sum\limits_{n = 1}^{\infty } {\left( {\frac{{k_{2n} {\text{e}}^{{k_{1n} \,t}} - k_{1n} {\text{e}}^{{k_{2n} \,t}} }}{{\left( {k_{1n} - k_{2n} } \right)\left( {\lambda^{2} + \omega_{n}^{2} } \right)}}} \right)} \cos \left( {\omega_{n} x} \right) $$

By using Eqs. (39) and (11), we get the dynamical temperature increment as follows:

40 $$ \theta \left( {x,t} \right) = \frac{{q_{0} }}{{2LK\lambda^{2} }} + \frac{\gamma \psi }{{2\lambda^{2} }} + \frac{{q_{0} }}{KL}\sum\limits_{n = 1}^{\infty } {\frac{{\left( {1 + \beta \omega_{n}^{2} } \right)}}{{\left( {\lambda^{2} + \omega_{n}^{2} } \right)}}} \cos \left( {\omega_{n} x} \right) + \left( {\frac{1}{{1 - \varepsilon \tau_{q} }}} \right)\left( {\frac{{q_{0} }}{{2LK\lambda^{2} }} + \frac{\gamma \psi }{{2\lambda^{2} }}} \right)\left[ {\varepsilon \tau_{q} {\text{e}}^{{\frac{ - t}{{\tau_{q} }}}} - {\text{e}}^{ - \varepsilon t} } \right] + \frac{{q_{0} }}{KL}\sum\limits_{n = 1}^{\infty } {\left( {1 - \beta \omega_{n}^{2} } \right)\left( {\frac{{k_{2n} {\text{e}}^{{k_{1n} \,t}} + k_{1n} {\text{e}}^{{k_{2n} \,t}} }}{{\left( {k_{1n} - k_{2n} } \right)\left( {\lambda^{2} + \omega_{n}^{2} } \right)}}} \right)} \cos \left( {\omega_{n} x} \right) $$

For the case of $\Delta < 0$, we have

41 $$ f_{1} \left( {k_{1n} ,t} \right) = {\text{e}}^{{\left( {k_{1n} \,t} \right)}} \cos \left( {\frac{{\sqrt { - \Delta_{n} } }}{2}\,t} \right),\quad f_{2} \left( {k_{2n} ,t} \right) = {\text{e}}^{{\left( {k_{2n} \,t} \right)}} \sin \left( {\frac{{\sqrt { - \Delta_{n} } }}{2}\,t} \right), $$

42 $$ \frac{{\partial f_{1} \left( {k_{1n} ,t} \right)}}{\partial \,t} = {\text{e}}^{{\left( {k_{1n} \,t} \right)}} \left[ {k_{1n} \,\cos \left( {\frac{{\sqrt { - \Delta_{n} } }}{2}\,t} \right) - \frac{{\sqrt { - \Delta } }}{2}\sin \left( {\frac{{\sqrt { - \Delta_{n} } }}{2}\,t} \right)} \right] $$

43 $$ \frac{{\partial f_{2} \left( {k_{2n} ,t} \right)}}{\partial t} = {\text{e}}^{{\left( {k_{2n} \,t} \right)}} \left[ {k_{2n} \sin \left( {\frac{{\sqrt { - \Delta_{n} } }}{2}\,t} \right) + \frac{{\sqrt { - \Delta_{n} } }}{2}\cos \left( {\frac{{\sqrt { - \Delta_{n} } }}{2}\,t} \right)} \right] $$

44 $$ f_{1} \left( {k_{1n} ,0} \right) = 1,\quad f_{2} \left( {k_{2n} ,0} \right) = 0,\quad n = 0,1,2,3 \ldots $$

45 $$ \frac{{\partial f_{1} \left( {k_{1n} ,0} \right)}}{\partial t} = k_{1n} ,\frac{{\partial f_{2} \left( {k_{2n} ,0} \right)}}{\partial t} = \frac{{\sqrt { - \Delta_{n} } }}{2},\quad n = 0,1,2,3 \ldots $$

$$ A_{10} = \frac{1}{{\tau_{q} }},\;A_{20} = \frac{\varepsilon }{{\tau_{q} }},\;\Delta_{0} = \left( {\frac{{\varepsilon \tau_{q} - 1}}{{\tau_{q} }}} \right)^{2} ,\,k_{10} = \frac{ - 1}{{\tau_{q} }},\;k_{20} = - \varepsilon $$

Applying the initial conditions (19), we obtain

46 $$ a_{0} + \sum\limits_{n = 1}^{\infty } {a_{n} } \cos \left( {\omega_{n} x} \right) = - \frac{{q_{0} }}{{2LK\lambda^{2} }} - \frac{\gamma \psi }{{2\lambda^{2} }} - \frac{{q_{0} }}{KL}\sum\limits_{n = 1}^{\infty } {\frac{1}{{\left( {\lambda^{2} + \omega_{n}^{2} } \right)}}} \cos \left( {\omega_{n} x} \right) $$

and

47 $$ \left( { - \frac{1}{{\tau_{q} }}} \right)a_{0} + \left( {\frac{{\tau_{q} - 1}}{{2\tau_{q} }}} \right)b_{0} + \sum\limits_{n = 1}^{\infty } {\left[ {a_{n} k_{1n} + b_{n} \frac{{\sqrt { - \Delta_{n} } }}{2}} \right]} \cos \left( {\omega_{n} x} \right) = 0 $$

Solving the above system, we obtain

48 $$ a_{0} = - \left( {\frac{{q_{0} }}{{2LK\lambda^{2} }} + \frac{\gamma \psi }{{2\lambda^{2} }}} \right),\quad b_{0} = - \left( {\frac{1}{{\tau_{q} - 1}}} \right)\left( {\frac{{q_{0} }}{{LK\lambda^{2} }} + \frac{\gamma \psi }{{\lambda^{2} }}} \right) $$

49 $$ a_{n} = - \frac{{q_{0} }}{{KL\left( {\lambda^{2} + \omega_{n}^{2} } \right)}},\quad b_{n} = \frac{{2k_{1n} q_{o} }}{{KL\left( {\lambda^{2} + \omega_{n}^{2} } \right)\sqrt { - \Delta_{n} } }} $$

Hence, the final solution of the heat conduction temperature increment in this case is:

50 $$ \begin{aligned} & \varphi \left( {x,t} \right) = \frac{{q_{0} }}{{2LK\lambda^{2} }} + \frac{\psi }{2} + \frac{{q_{0} }}{KL}\sum\limits_{n = 1}^{\infty } {\frac{1}{{\left( {\lambda^{2} + \omega_{n}^{2} } \right)}}} \cos \left( {\omega_{n} x} \right) - \left( {\frac{{q_{0} }}{{2LK\lambda^{2} }} + \frac{\gamma \psi }{{2\lambda^{2} }}} \right){\text{e}}^{{\frac{ - t}{{\tau_{q} }}}} \cos \left( {\frac{{\sqrt { - \Delta_{0} } }}{2}\,t} \right) - \left( {\frac{1}{{\tau_{q} - 1}}} \right)\left( {\frac{{q_{0} }}{{LK\lambda^{2} }} + \frac{\gamma \psi }{{\lambda^{2} }}} \right){\text{e}}^{ - t} \sin \left( {\frac{{\sqrt { - \Delta_{0} } }}{2}\,t} \right) \\ & \quad \quad \quad \quad + \frac{{q_{0} }}{KL}\,\,\sum\limits_{n = 1}^{\infty } {\left[ {{\text{e}}^{{\left( {k_{1n} \,t} \right)}} \cos \left( {\frac{{\sqrt { - \Delta_{n} } }}{2}\,t} \right) + \frac{{2k_{1n} }}{{\sqrt { - \Delta_{n} } }}{\text{e}}^{{\left( {k_{2n} \,t} \right)}} \sin \left( {\frac{{\sqrt { - \Delta_{n} } }}{2}\,t} \right)} \right]} \frac{{\cos \left( {\omega_{n} x} \right)}}{{\left( {\lambda^{2} + \omega_{n}^{2} } \right)}} \\ \end{aligned} $$

By using Eqs. (50) and (11), we get the dynamical temperature increment in this case as follows:

51 $$ \begin{aligned} & \theta \left( {x,t} \right) = \frac{{q_{0} }}{{2LK\lambda^{2} }} + \frac{\psi }{2} + \frac{{q_{0} }}{KL}\sum\limits_{n = 1}^{\infty } {\frac{{\left( {1 + \omega_{n}^{2} \beta } \right)}}{{\left( {\lambda^{2} + \omega_{n}^{2} } \right)}}} \cos \left( {\omega_{n} x} \right) - \left( {\frac{{q_{0} }}{{2LK\lambda^{2} }} + \frac{\gamma \psi }{{2\lambda^{2} }}} \right){\text{e}}^{{\frac{ - t}{{\tau_{q} }}}} \cos \left( {\frac{{\sqrt { - \Delta_{0} } }}{2}\,t} \right) - \left( {\frac{1}{{\tau_{q} - 1}}} \right)\left( {\frac{{q_{0} }}{{LK\lambda^{2} }} + \frac{\gamma \psi }{{\lambda^{2} }}} \right){\text{e}}^{ - t} \sin \left( {\frac{{\sqrt { - \Delta_{0} } }}{2}\,t} \right) \\ & \quad \quad \quad \quad + \frac{{q_{0} }}{KL}\,\,\sum\limits_{n = 1}^{\infty } {\left[ {{\text{e}}^{{\left( {k_{1n} \,t} \right)}} \cos \left( {\frac{{\sqrt { - \Delta_{n} } }}{2}\,t} \right) + \frac{{2k_{1n} }}{{\sqrt { - \Delta_{n} } }}{\text{e}}^{{\left( {k_{2n} \,t} \right)}} \sin \left( {\frac{{\sqrt { - \Delta_{n} } }}{2}t} \right)} \right]} \frac{{\left( {1 + \omega_{n}^{2} \beta } \right)\cos \left( {\omega_{n} x} \right)}}{{\left( {\lambda^{2} + \omega_{n}^{2} } \right)}} \\ \end{aligned} $$

## Results

In this study, the temperature distribution through skin tissue is investigated for two models (One temperature, two temperature) of bioheat transfer for constant heat flux condition on the skin surface. The values of the relevant thermal parameters used in the present calculations are in Table 1 as follows:

**Table 1:** The material properties of the skin tissue.

Parameter	Unit	Skin	Parameter	Unit	Skin
$K$	W/m°C	0.215	$W_{b}$	ml/Cm	0.00052
$\rho$	kg/m^3^	1000	$T_{b}$	°C	37
$\rho_{b}$	kg/m^3^	1060	$\tau_{T}$	s	10.0
$C$	J/kg°C	4187	$\tau_{q}$	s	20.0
$C_{b}$	J/kg°C	3800	L	m	0.006

## Discussions

**Figure 2:**
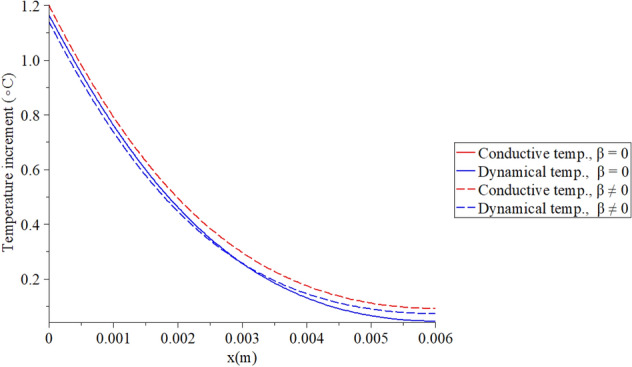
The temperature increment when $t = 100\,({\text{s}}),\,\,\tau_{T} = 10\,({\text{s}}),\,\,\tau_{q} = 20\,({\text{s}})$ and $q_{0} = 100\;({\text{W/m}}^{{2}} )$.

**Figure 3:**
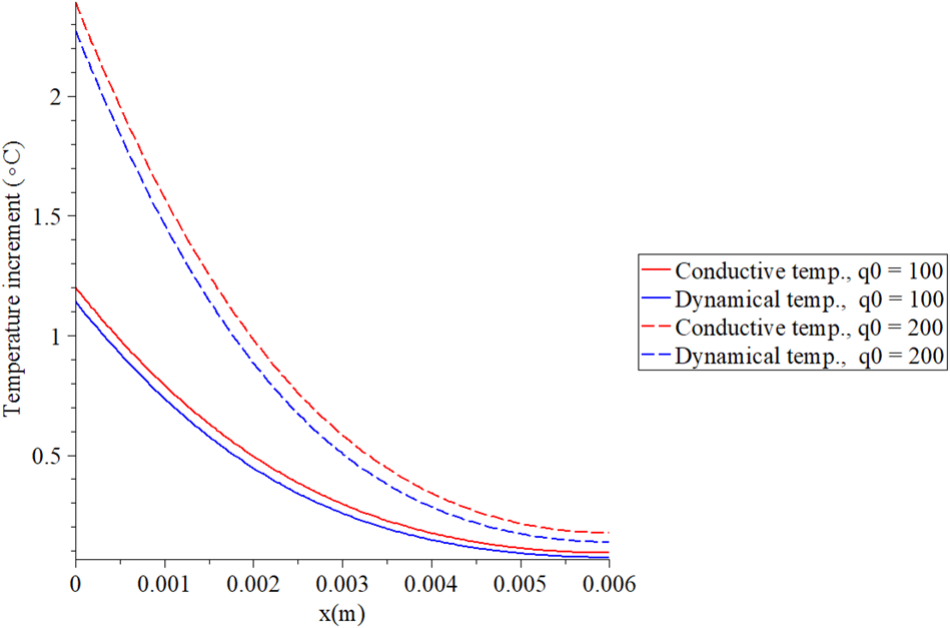
The temperature increment when $t = 100\,\left( {\text{s}} \right),\,\,\tau_{T} = 10\,\left( {\text{s}} \right),\,\,\tau_{q} = 20\,\left( {\text{s}} \right)$ and various values of the surface heat flux $q_{0} \,\left( {{\text{W/m}}^{{2}} } \right)$ based on the two-temperature model.

**Figure 4:**
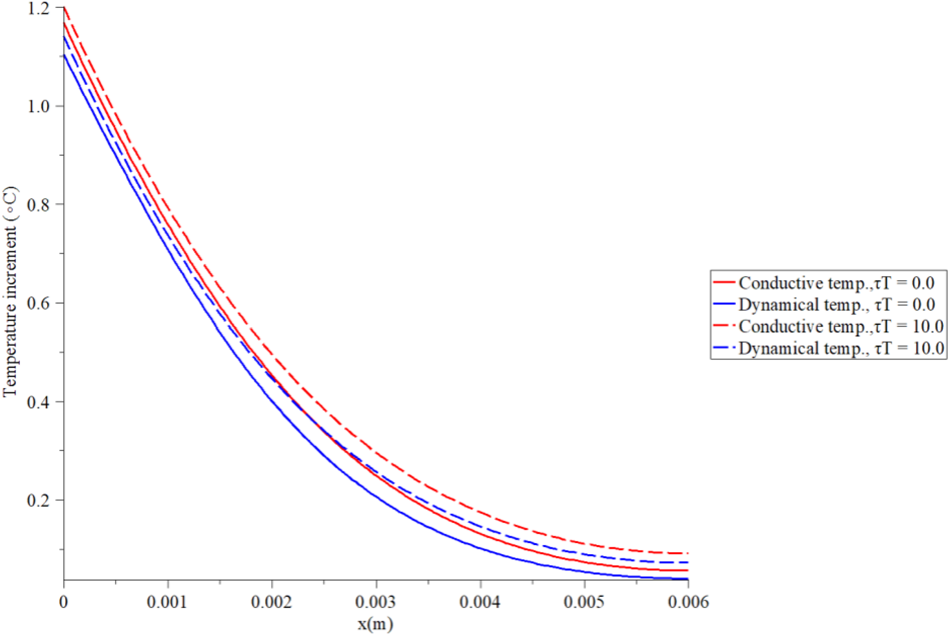
The temperature increment when $t = 100\,\left( {\text{s}} \right),\,\,q_{0} = 100\,\,\left( {{\text{W/m}}^{{2}} } \right),\,\,\tau_{q} = 20\,\left( {\text{s}} \right)$ and various values $\tau_{T} \left( {\text{s}} \right)$ based on the two-temperature model.

**Figure 5:**
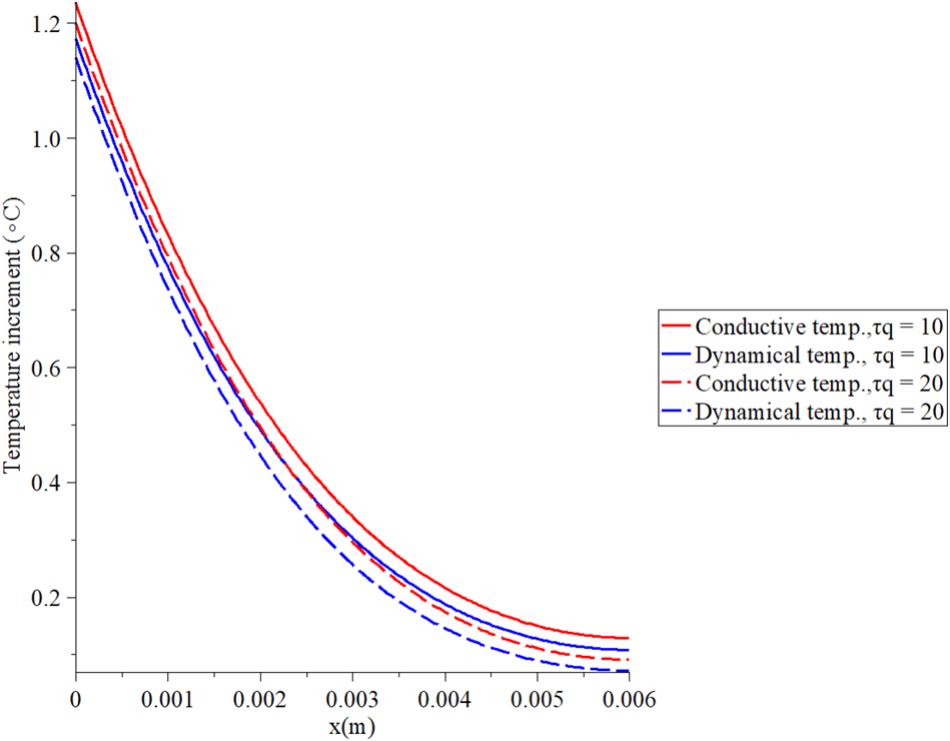
The temperature increment when $t = 100\,\left( {\text{s}} \right),\,\,q_{0} = 100\,\,\left( {{\text{W/m}}^{{2}} } \right),\,\,\tau_{T} = 10\,\left( {\text{s}} \right)$ and various values $\tau_{q} \left( {\text{s}} \right)$ based on the two-temperature model.

Figures 2, 3, 4, 5 represent the conductive and dynamical temperature increment with respect to a wide range of skin distance *x* $(0 \le x \le 0.006\,\,{\text{m}})$ and at constant instance time $t = 100({\text{s}})$.

Figure 2 shows that the two temperature parameter $\beta$ has a significant effect on the temperature increment distribution. The red line represents the conductive temperature distribution, while the blue line represents the dynamical temperature distribution. The conductive temperature increment and dynamical temperature increment have the same behavior but with different values because of the effect of the two-temperature parameter. In the context of the one-temperature model $\beta = 0$, the conductive and dynamical temperature increments are the same, and they appear in the solid blue curve. It is noted that an increase in the value of the two-temperature parameter leads to an increase in the value of conductive temperature increment and a decrease in the value of dynamical temperature increment. We noticed that thermal waves in the context of the one-temperature model have vanished before the thermal waves in the context of the two-temperature model.

Figure 3 shows that the conductive and dynamical temperature increment distributions when $t = 100\;({\text{s)}},\,\,\tau_{T} = 10\;({\text{s}}),\,\,\tau_{q} = 20\;({\text{s}})$ and various values of the surface heat flux $q_{0} \,({\text{W/m}}^{{2}} ) = \left( {100,200} \right)$ to stand on its effects. It is noted that the value of the heat flux on the surface of the skin tissue has a significant impact on the conductive and dynamical temperature increment distributions. An increase in the value of the heat flux leads to an increase in the value of the conductive and dynamical temperature increment distributions. A smaller value of the surface heat flux makes the temperature more closed to zero value at the end of the skin length.

Figure 4 shows that the conductive and dynamical temperature increment distributions when $t = 100\;({\text{s}}),\,q_{0} = 100\;({\text{W/m}}^{{2}} ),\,\,\tau_{q} = 20\;({\text{s)}}$ and various values of the second relaxation time parameter $\tau_{T} ({\text{s}}) = (0.0,10.0)$ to stand on its effects. It is noted that the value of the second relaxation time parameter has a significant impact on the conductive and dynamical temperature increment distributions. An increase in the value of the second relaxation time parameter leads to an increase in the value of the conductive and dynamical temperature increment distributions. The zero value of the first relaxation time parameter $\tau_{T}$ (temperature lag-time) makes the thermal waves more closed to the zero value at the end of the skin length.

Figure 5 shows that the conductive and dynamical temperature increment distributions when $t = 100\,{\text{(s}}),\,\,q_{0} = 100\,\,({\text{W/m}}^{{2}} ),\,\,\tau_{T} = 10\,({\text{s}})$ and various values of the first relaxation time parameter $\tau_{q} \left( {\text{s}} \right) = \left( {10,20} \right)$ to stand on its effects. It is noted that the value of the first relaxation time $\tau_{q}$ parameter has noticeable effects on the conductive and dynamical temperature increment distributions. An increase in the value of the second relaxation time parameter $\tau_{q}$ leads to a decrease in the value of the conductive and dynamical temperature increment distributions. Increasing the value of the second relaxation time parameter $\tau_{q}$(temperature gradient lag-time) makes the thermal waves more closed to the zero value at the end of the skin length.

**Figure 6:**
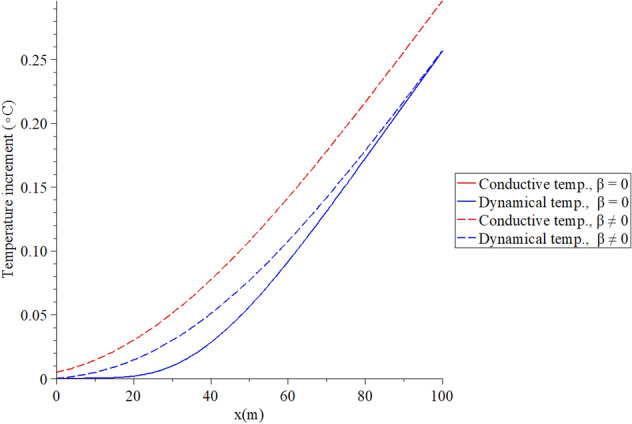
The temperature increment when $x = 0.003\,\left( {\text{m}} \right),\,\,\tau_{T} = 10\,\left( {\text{s}} \right),\,\,\tau_{q} = 20\,\left( {\text{s}} \right)$ and $q_{0} = 100\,\,\left( {W/m^{2} } \right)$.

**Figure 7:**
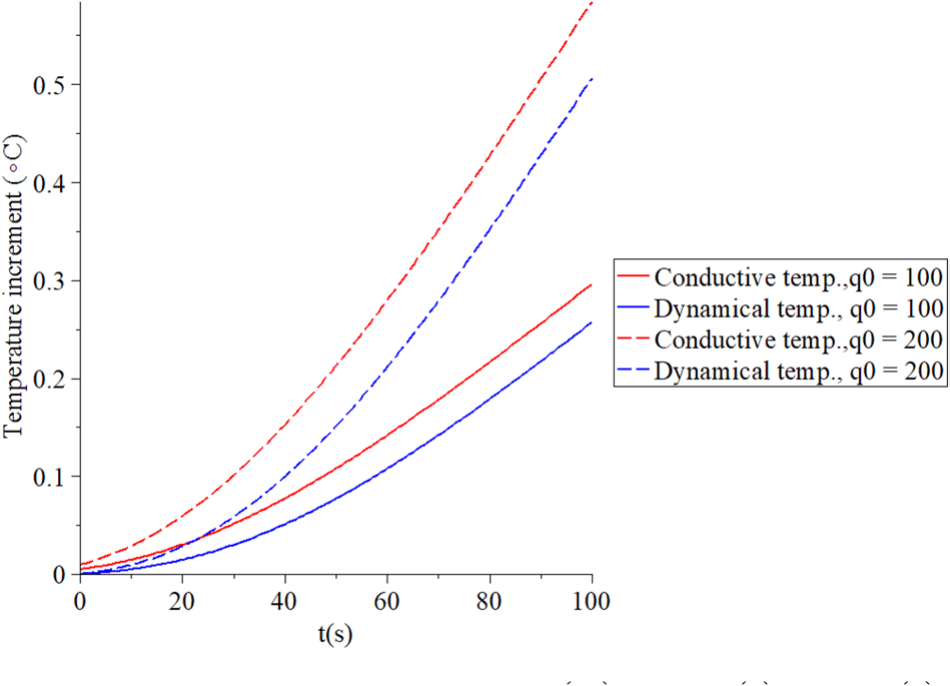
The temperature increment when $x = 0.003\,\left( {\text{m}} \right),\,\,\tau_{T} = 10\,\left( {\text{s}} \right),\,\,\tau_{q} = 20\,\left( {\text{s}} \right)$ and various values of the surface heat flux $q_{0} \,\left( {{\text{W/m}}^{{2}} } \right)$ based on the two-temperature model.

**Figure 8:**
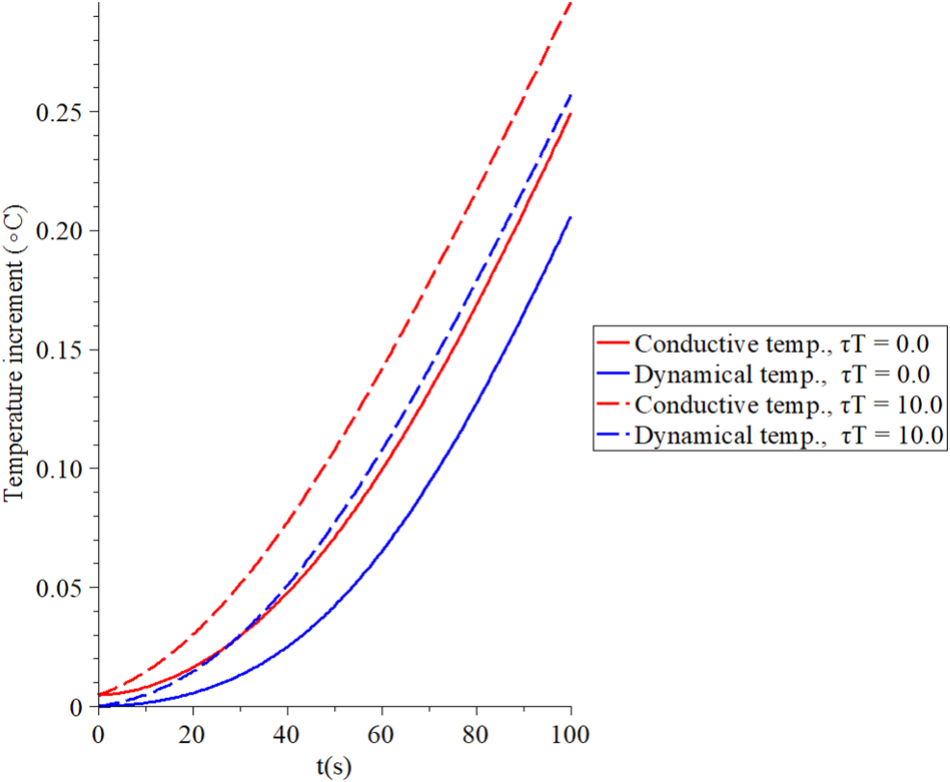
The temperature increment when $x = 0.003\,\left( {\text{m}} \right),\,\,q_{0} = 100\,\,\left( {{\text{W/m}}^{{2}} } \right),\,\,\tau_{q} = 20\,\left( {\text{s}} \right)$ and various values $\tau_{T} \left( {\text{s}} \right)$ based on the two-temperature model.

**Figure 9:**
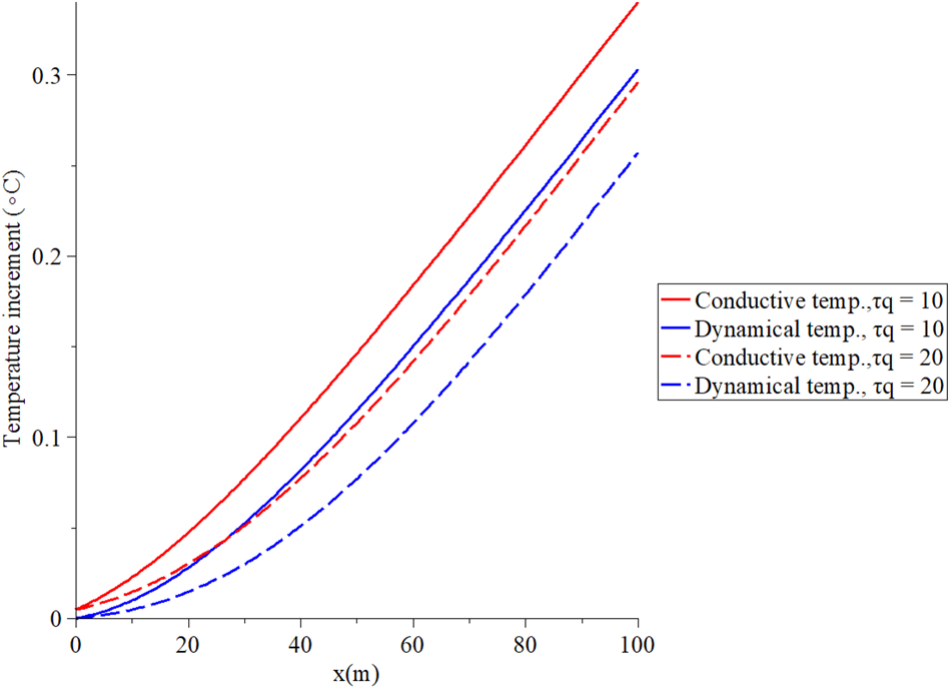
The temperature increment when $x = 0.003\,\left( {\text{m}} \right),\,\,q_{0} = 100\,\,\left( {{\text{W/m}}^{{2}} } \right),\,\,\tau_{T} = 10\,\left( {\text{s}} \right)$ and various values $\tau_{q} \left( {\text{s}} \right)$ based on the two-temperature model.

Figures 6, 7, 8, 9 represent the conductive and dynamical temperature increment with respect to a wide range of time *x* $\left( {0 \le t \le 100\,\,{\text{s}}} \right)$ and at a constant distance $x = 0.003\left( {\text{m}} \right)$.

Figure 6 shows that the two temperature parameter $\beta$ has a significant effect on the temperature increment distribution. The conductive temperature increment and dynamical temperature increment have the same behavior but with different values because of the impact of the two-temperature parameter. In the context of the one-temperature model $\beta = 0$, the conductive and dynamical temperature increments are the same, and the solid blue curve represents them. It is noted that an increase in the value of the two-temperature parameter leads to an increase in the value of conductive temperature increment and a decrease in the value of dynamical temperature increment.

Figure 7 shows that the conductive and dynamical temperature increment distributions when $x = 0.003\,\left( {\text{m}} \right),\,\,\tau_{T} = 10\,\left( {\text{s}} \right),\,\,\tau_{q} = 20\,\left( {\text{s}} \right)$ and various values of the surface heat flux $q_{0} \,\left( {W/m^{2} } \right) = \left( {100,200} \right)$ to stand on its effects. It is noted that the value of the heat flux on the surface of the skin tissue has a significant impact on the conductive and dynamical temperature increment distributions. An increase in the value of the heat flux leads to an increase in the value of the conductive and dynamical temperature increment distributions.

Figure 8 shows that the conductive and dynamical temperature increment distributions when $x = 0.003\,\left( {\text{m}} \right),\,\,q_{0} = 100\,\,\left( {{\text{W/m}}^{{2}} } \right),\,\,\tau_{q} = 20\,\left( {\text{s}} \right)$ and various values of the second relaxation time parameter $\tau_{T} \left( {\text{s}} \right) = \left( {0.0,10.0} \right)$ to stand on its effects. It is noted that the value of the second relaxation time parameter has a significant impact on the conductive and dynamical temperature increment distributions. An increase in the value of the second relaxation time parameter leads to an increase in the value of the conductive and dynamical temperature increment distributions.

Figure 9 shows that the conductive and dynamical temperature increment distributions when $x\left( {\text{m}} \right) = 0.003\,,\,\,q_{0} \left( {{\text{W/m}}^{{2}} } \right) = 100\,\,,\,\,\tau_{T} \left( {\text{s}} \right) = 10\,$ and various values of the second relaxation time parameter $\tau_{q} \left( {\text{s}} \right) = \left( {0.0,10.0} \right)$ to stand on its effects. It is noted that the value of the first relaxation time parameter has a significant impact on the conductive and dynamical temperature increment distributions. An increase in the value of the first relaxation time parameter leads to a decrease in the value of the conductive and dynamical temperature increment distributions.

**Figure 10:**
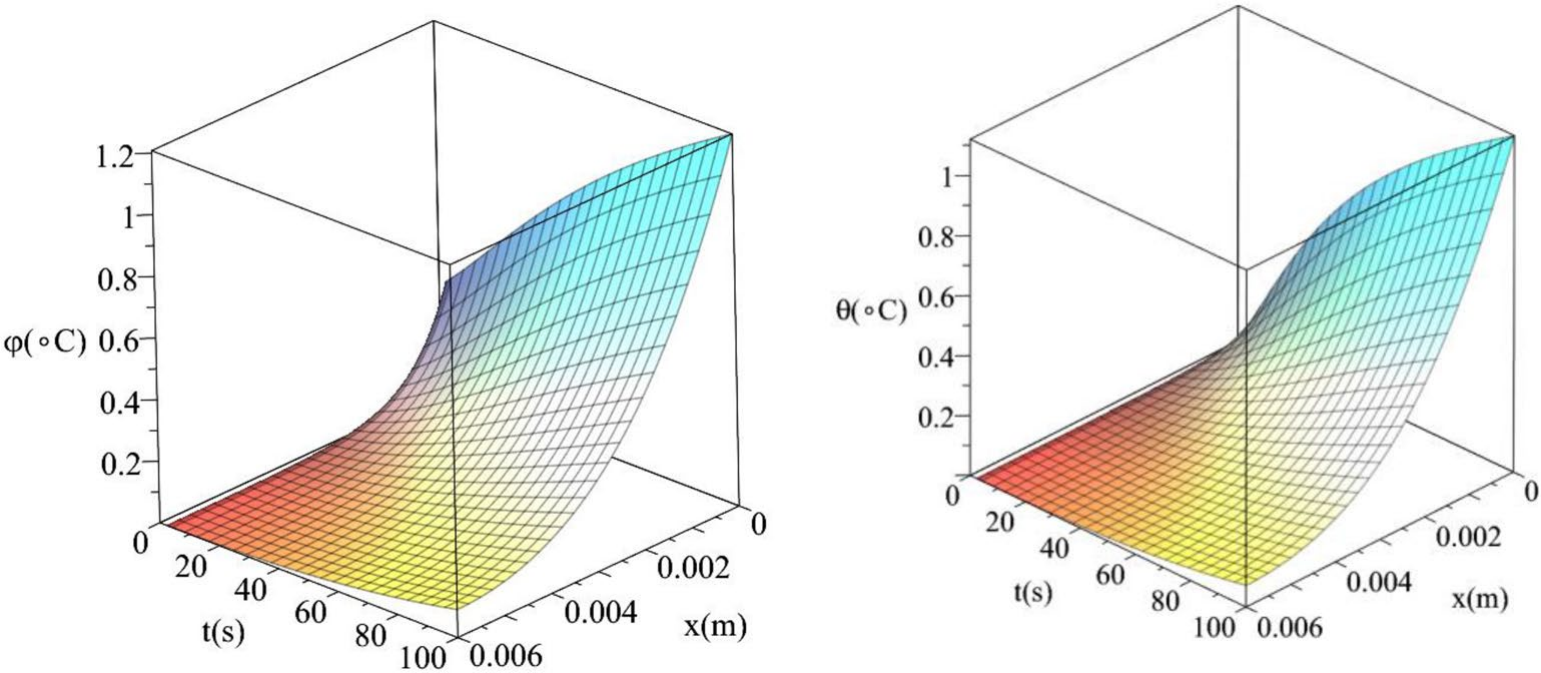
The conductive and dynamical temperature increment when $q_{0} = 100\left( {{\text{W/m}}^{{2}} } \right)$ based on the two-temperature model.

**Figure 11:**
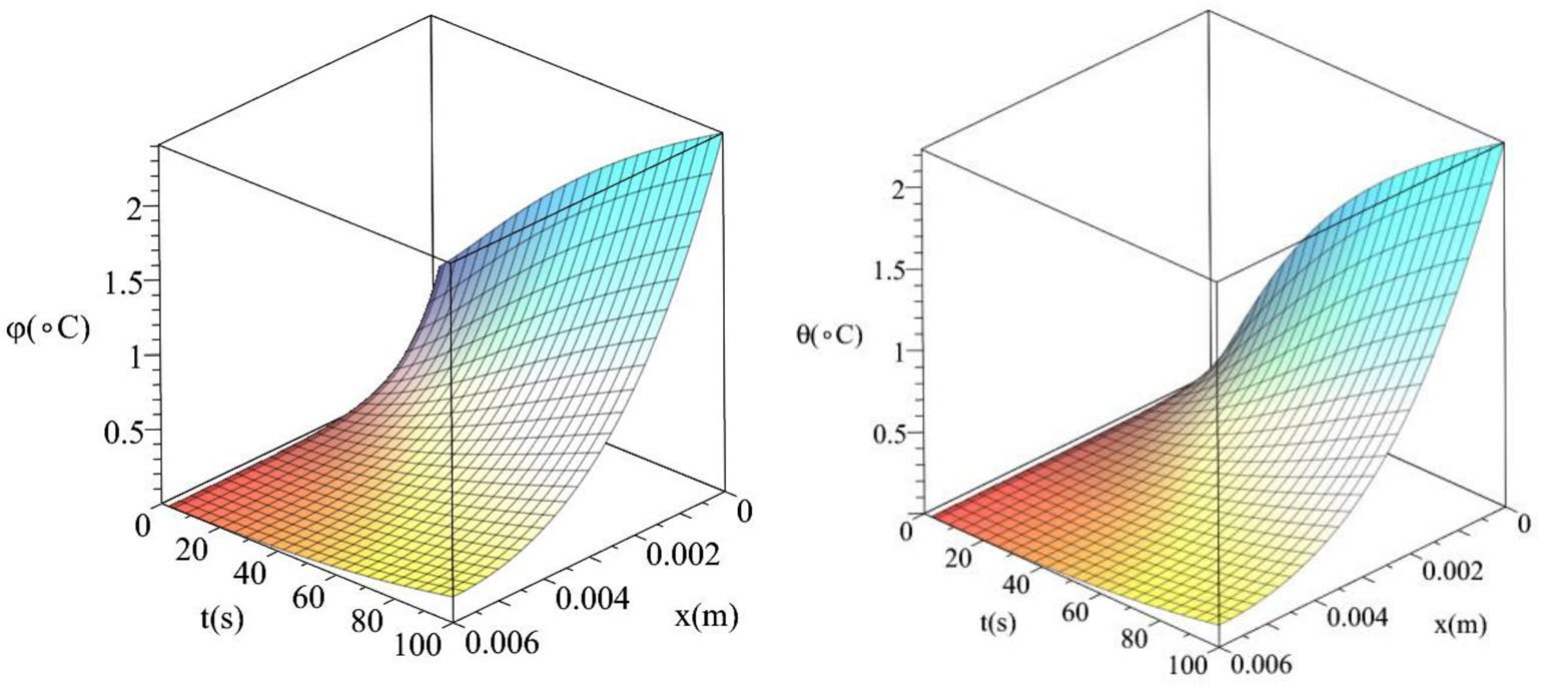
The conductive and dynamical temperature increment when $q_{0} = 200\left( {{\text{W/m}}^{{2}} } \right)$ based on the two-temperature model.

Figures 10 and 11 show the differences between the conductive temperature increment and dynamical temperature increment with respect to a wide range of time *t* $\left( {0 \le t \le 100\left( {\text{s}} \right)\,} \right)$ and distance $x\left( {0 \le x \le 0.006\left( {\text{m}} \right)\,} \right)$. Figure 8 represents the conductive temperature increment and dynamical temperature increment when $q_{0} \left( {{\text{W/m}}^{{2}} } \right) = 100$ and Fig. 9 when $q_{0} \left( {{\text{W/m}}^{{2}} } \right) = 200$. These two figures agree with the results in Figs. 3 and 6 and confirm that the value of the heat flux on the surface of the skin tissue has significant effects on the conductive and dynamical temperature increment distributions.

**Figure 12:**
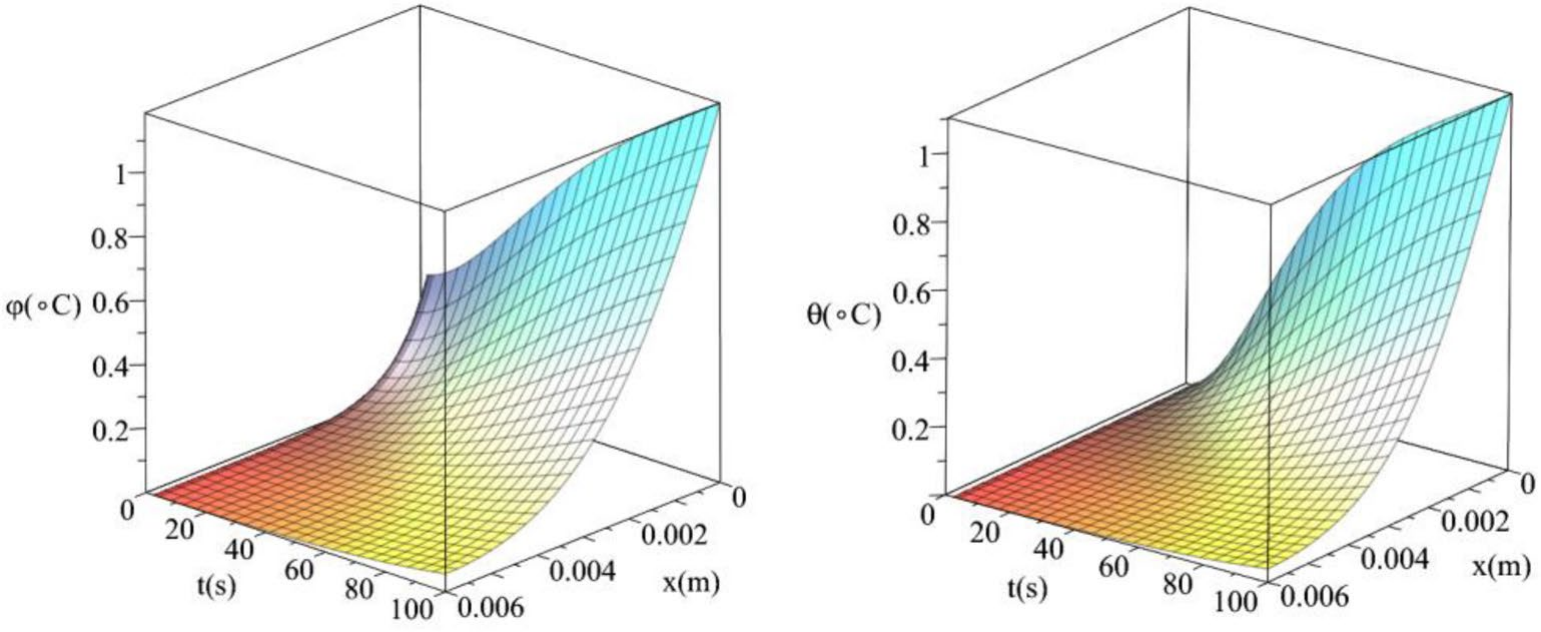
The conductive and dynamical temperature increment when $\tau_{T} \left( {\text{s}} \right) = 0.0$ based on the two-temperature model.

**Figure 13:**
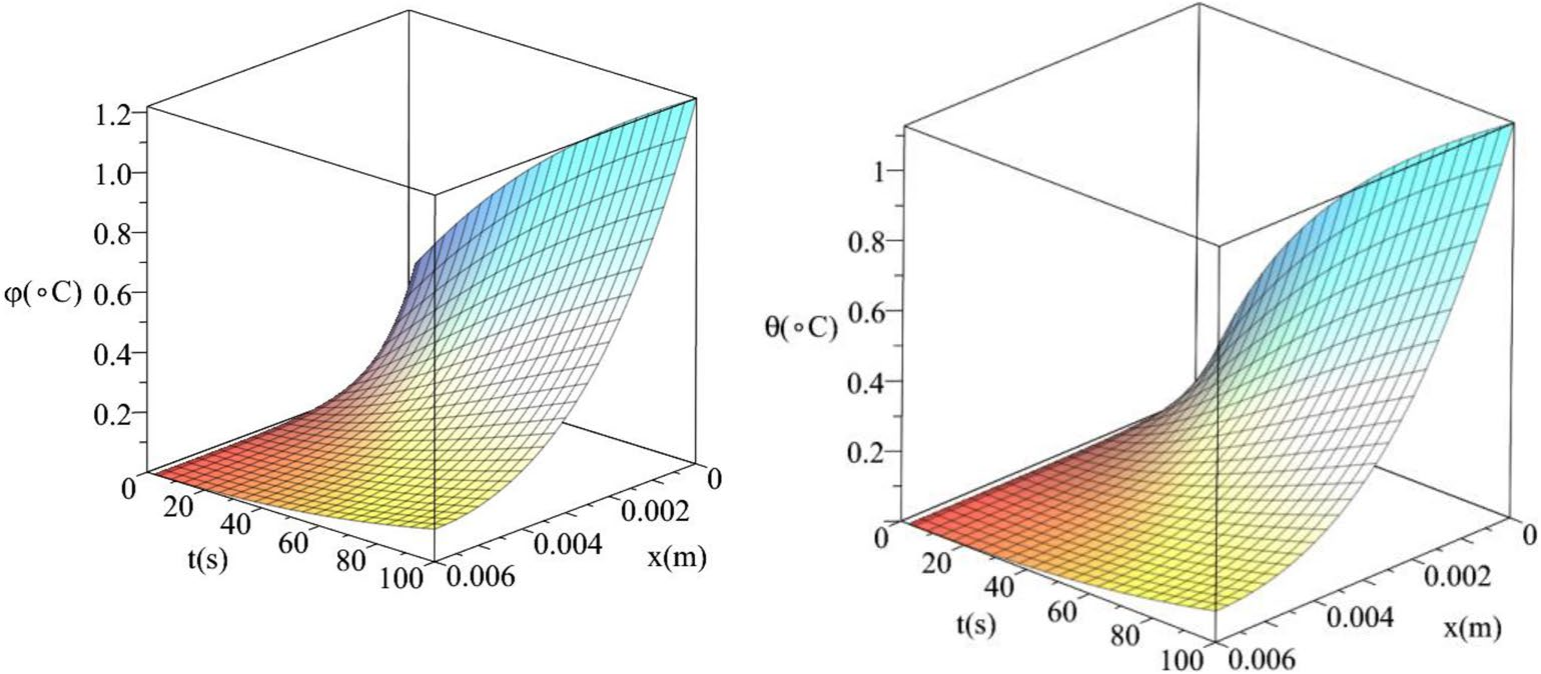
The conductive and dynamical temperature increment when $\tau_{T} \left( {\text{s}} \right) = 15$ based on the two-temperature model.

Figures 12 and 13 show the differences between the conductive temperature increment and dynamical temperature increment with respect to a wide range of time *t* $\left( {0 \le t \le 100\left( {\text{s}} \right)\,} \right)$ and distance $x\left( {0 \le x \le 0.006\left( {\text{m}} \right)\,} \right)$ for various values of the second relaxation time parameter $\tau_{T} \left( {\text{s}} \right) = \left( {0.0,10.0} \right)$ This two figures agree with the results in the Figs. 4 and 7 and confirm that the value of the second relaxation time parameter has significant effects on the conductive and dynamical temperature increment distributions.

**Figure 14:**
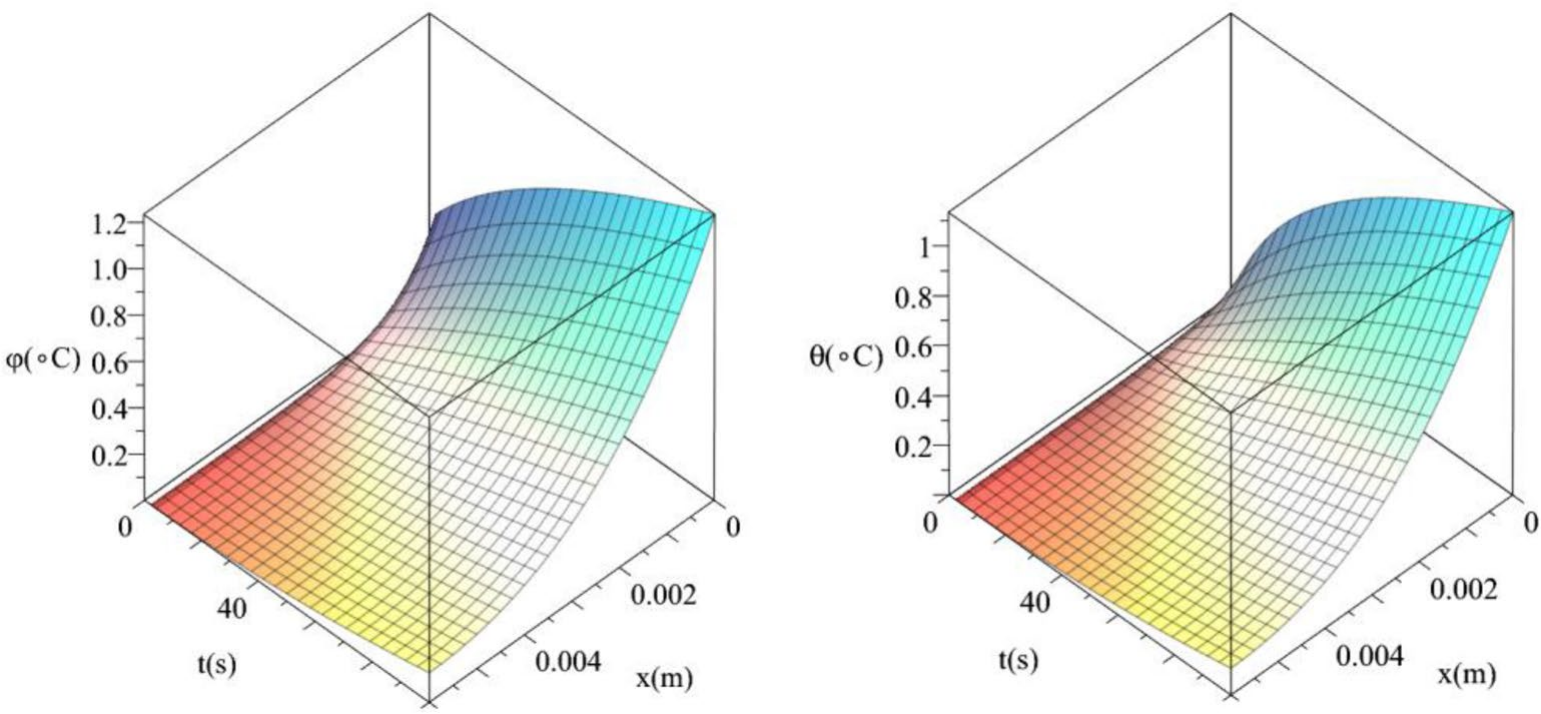
The conductive and dynamical temperature increment when $\tau_{q} \left( {\text{s}} \right) = 10$.

**Figure 15:**
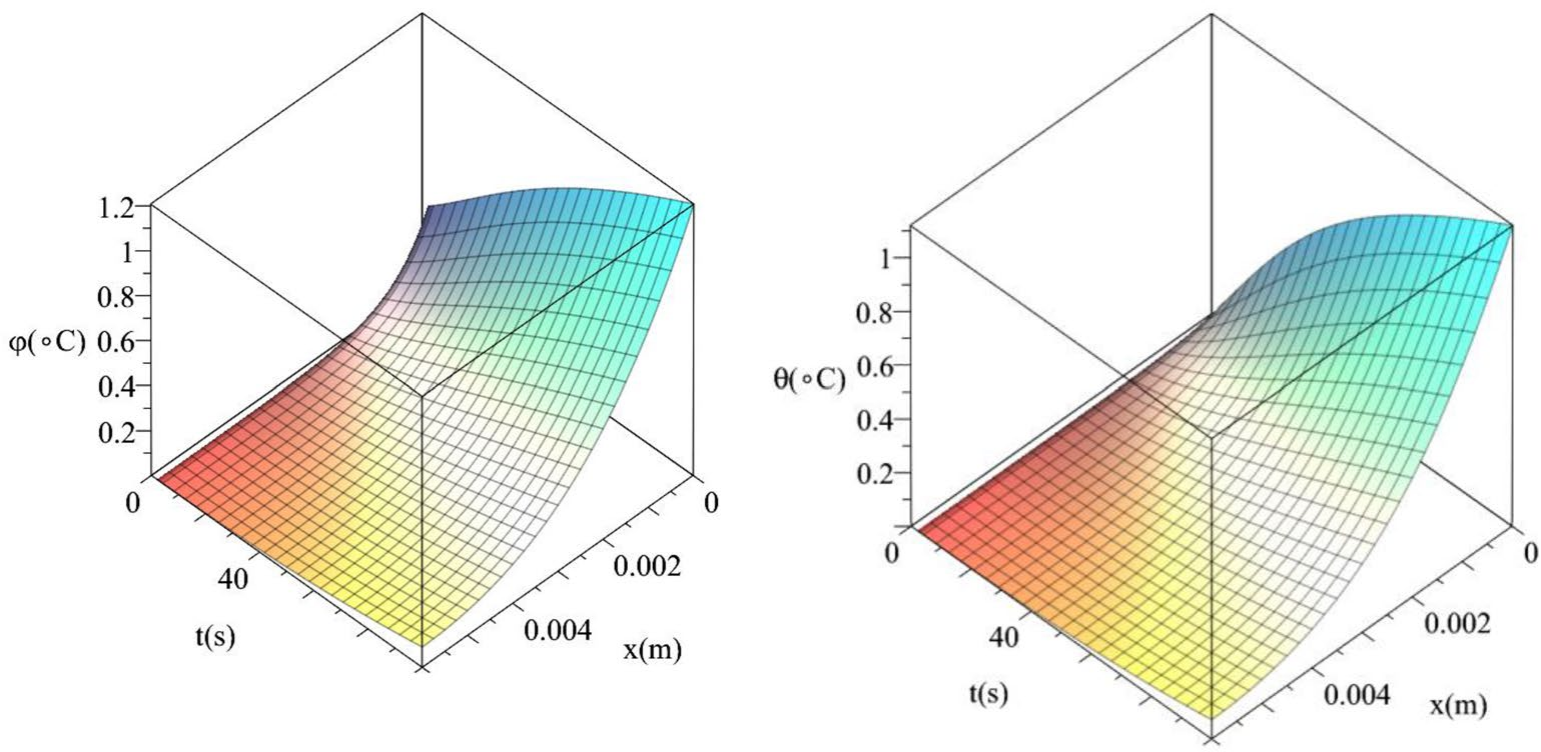
The conductive and dynamical temperature increment when $\tau_{q} \left( {\text{s}} \right) = 20$.

Figures 14 and 15 show the differences between the conductive temperature increment and dynamical temperature increment with respect to a wide range of time *t* $\left( {0 \le t \le 100\left( {\text{s}} \right)\,} \right)$ and distance $x\left( {0 \le x \le 0.006\left( {\text{m}} \right)\,} \right)$ for various values of the first relaxation time parameter $\tau_{q} \left( {\text{s}} \right) = \left( {10,20} \right)$. These two figures agree with the results in Figs. 5 and 9 and confirm that the value of the first relaxation time parameter has significant effects on the conductive and dynamical temperature increment distributions.

Here, we should refer to the agreement of the results of this research with the results of other researches in order to confirm the validity of the current results and the validity of the proposed mathematical model as well as the method of solution.

**Figure 16:**
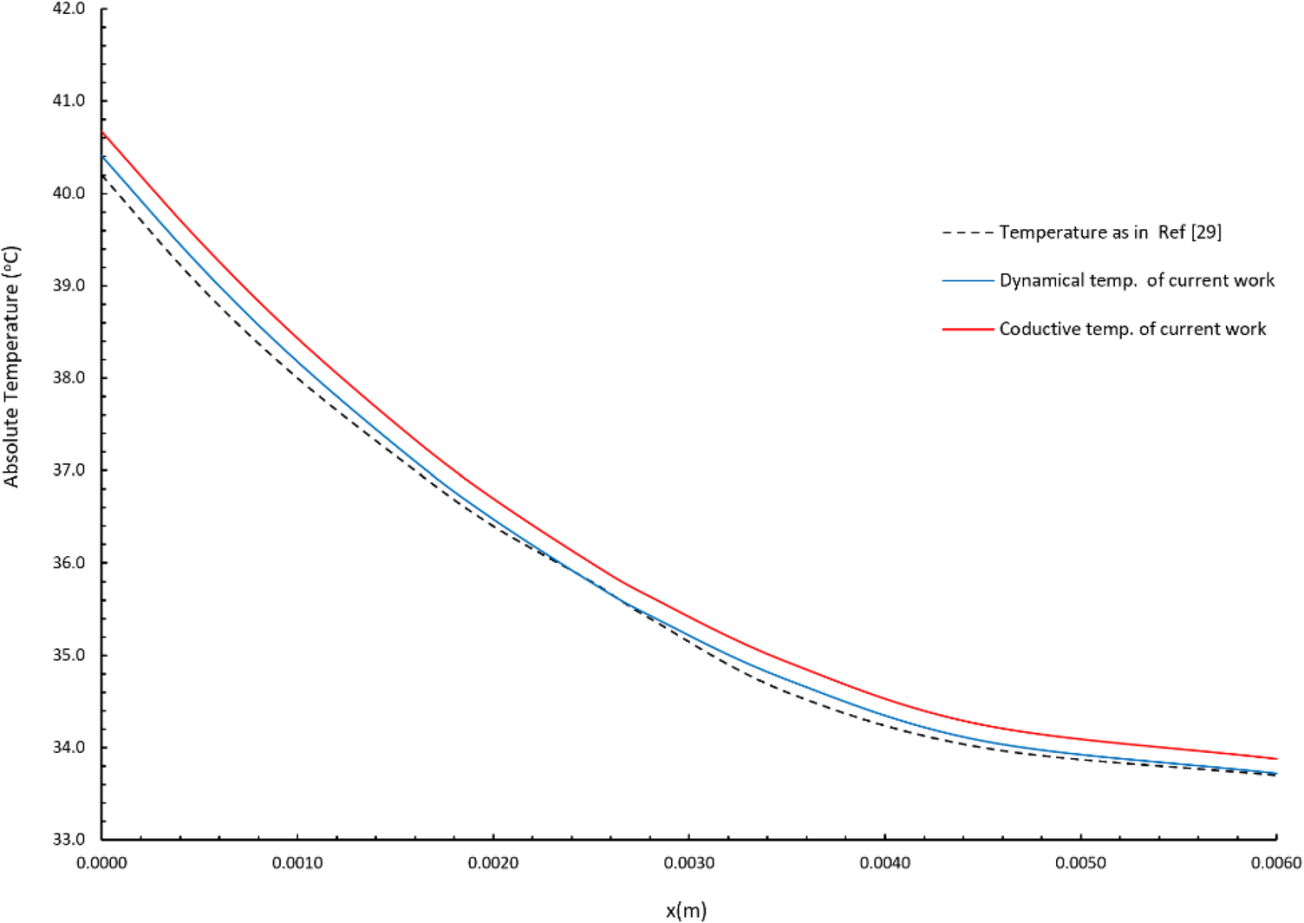
The absolute conductive and dynamical temperatures of the current model against Kundu and Dewanjee^29^ when $\rho_{b} W_{b} = 0.5\,\,{\text{kg/m}}^{{3}} {\text{s}},\,\,\tau_{T} = 0,\,\,\tau_{q} = 20\,{\text{s}}$, ${\text{K } = \text{ 0}}{\text{.2 W/m }}^{{\text{o}}} {\text{C}}$, $\,t = 160\,{\text{s}}$, $\,{\text{q}}_{0} = 500\,{\text{W/m}}^{{2}}$.

The current results agree with the results of Youssef and Alghamdi^28,37^. Moreover, the current resluts agree with the result in figuer 6 of Kundu and Dewanjee^29^ for the case of boundary condition of cas (b). To verify our results with the results in Kundu and Dewanjee^29^, we represented Fig. 16 in which we use the same values of the parameters in Kundu and Dewanjee^29^ as follows:

$$ \rho_{b} W_{b} = 0.5\,{\text{kg/m}}^{{3}} {\text{s}},\;\tau_{T} = 0,\,\,\tau_{q} = 20\,{\text{s,}}\;{\text{K } = \text{ 0}}{\text{.2 W/m }}^{{\text{o}}} {\text{C,}}\;t = 160\,{\text{s,}}\,\,{\text{q}}_{0} = 500\,{\text{W/m}}^{{2}} $$

and calculating the absolut values of the dynamical and conductive temperature by adding $T_{b} = 37\,^{{\text{o}}} {\text{C}}$ to the increments as follwos:

$$ T_{D} = \left( {\theta + T_{b} } \right),\,\,\,T_{C} = \left( {\varphi + T_{b} } \right) $$

Figure 16 show that the current result of the absolute dynamical and conductive temperature are close to the values of the absolute temperature in Kundu and Dewanjee^29^. Thus, we have a guarantee that the results of the current research are correct and verified.

## Conclusion

- The dual-phase lag time parameters $\tau_{T} ,\,\,\tau_{q}$ have significant effects on the conductive and the dynamical temperature increment of the skin tissue.
- The initial heat flux applying on the surface of the skin tissue has significant on the conductive and the dynamical temperature increment.
- The two-temperature parameter has a significant effect on the conductive and the dynamical temperature increment.
- The Two-temperature dual-phase-lag (TTDPL) bioheat transfer model is a successful model to simulate the thermal behavior of the skin tissue.
- The results in the current work agree with the results of Kundu and Dewanjee^29^. Therefore, the two-temperature dual-phase-lag (TTDPL) bioheat transfer model be a succsessful model to study the bioheat transfer of a skin tissue and supplies us with exact solutions.

## Appendix

Subistituting from Eqs. (14) into (10), we have

52 $$ \left[ {\beta \rho C\tau_{q} \frac{{\partial^{2} }}{{\partial t^{2} }}\frac{{\partial^{2} }}{{\partial x^{2} }}\, + \left( {K\tau_{T} + \beta \rho C\, + \beta \tau_{q} w_{b} C_{b} \rho_{b} } \right)\frac{\partial }{\partial t}\frac{{\partial^{2} }}{{\partial x^{2} }} + \left( {\beta w_{b} C_{b} \rho_{b} + K} \right)\frac{{\partial^{2} }}{{\partial x^{2} }}} \right]\left[ {\phi_{1} \left( {x,t} \right) + \phi_{2} \left( x \right)} \right] = \left( {\tau_{q} \rho C\frac{{\partial^{2} }}{{\partial t^{2} }} + \left( {\rho C + \tau_{q} w_{b} C_{b} \rho_{b} } \right)\frac{\partial }{\partial t} + w_{b} C_{b} \rho_{b} } \right)\left[ {\phi_{1} \left( {x,t} \right) + \phi_{2} \left( x \right)} \right] - \psi $$

which gives

53 $$ \left[ {\beta \rho C\tau_{q} \frac{{\partial^{2} }}{{\partial t^{2} }}\frac{{\partial^{2} \phi_{1} }}{{\partial x^{2} }}\, + \left( {K\tau_{T} + \beta \rho C\, + \beta \tau_{q} w_{b} C_{b} \rho_{b} } \right)\frac{\partial }{\partial t}\frac{{\partial^{2} \phi_{1} }}{{\partial x^{2} }} + \left( {\beta w_{b} C_{b} \rho_{b} + K} \right)\frac{{\partial^{2} \phi_{1} }}{{\partial x^{2} }}} \right] = \left( {\beta w_{b} C_{b} \rho_{b} + K} \right)\frac{{d^{2} \phi_{2} }}{{dx^{2} }} + \,\,\left( {\tau_{q} \rho C\frac{{\partial^{2} \phi_{1} }}{{\partial t^{2} }} + \left( {\rho C + \tau_{q} w_{b} C_{b} \rho_{b} } \right)\frac{{\partial \phi_{1} }}{\partial t} + w_{b} C_{b} \rho_{b} \phi_{1} } \right) + w_{b} C_{b} \rho_{b} \phi_{2} - \psi $$

The Eq. (53) could be separated into two differential equation, one is a partial differential equation of two variables *x* and *t*, while the second one is ordinary differential equation of one variable *x* only as follows*:*

54 $$ \left[ {\beta \rho C\tau_{q} \frac{{\partial^{2} }}{{\partial t^{2} }}\frac{{\partial^{2} \phi_{1} }}{{\partial x^{2} }}\, + \left( {K\tau_{T} + \beta \rho C\, + \beta \tau_{q} w_{b} C_{b} \rho_{b} } \right)\frac{\partial }{\partial t}\frac{{\partial^{2} \phi_{1} }}{{\partial x^{2} }} + \left( {\beta w_{b} C_{b} \rho_{b} + K} \right)\frac{{\partial^{2} \phi_{1} }}{{\partial x^{2} }}} \right] = \left( {\tau_{q} \rho C\frac{{\partial^{2} \phi_{1} }}{{\partial t^{2} }} + \left( {\rho C + \tau_{q} w_{b} C_{b} \rho_{b} } \right)\frac{{\partial \phi_{1} }}{\partial t} + w_{b} C_{b} \rho_{b} \phi_{1} } \right) $$

and

55 $$ \,\left( {\beta w_{b} C_{b} \rho_{b} + K} \right)\frac{{d^{2} \phi_{2} }}{{dx^{2} }} = \,w_{b} C_{b} \rho_{b} \phi_{2} - \psi $$

The differentia Eq. (55)is the steady-state part and it takes the form

56 $$ \left( {\frac{{d^{2} }}{{dx^{2} }} - \lambda^{2} } \right)\phi_{2} \left( x \right) = - \gamma \,\psi $$

where $$\,\,\,\lambda^{2} = \,\frac{{w_{b} C_{b} \rho_{b} }}{{\left( {\beta w_{b} C_{b} \rho_{b} + K} \right)}}\,\, > 0,\,\,\gamma = \frac{1}{{\left( {\beta w_{b} C_{b} \rho_{b} + K} \right)}} > 0$$, and $$\psi = Q_{met}$$.

The boundary conditions for the steady-state (17) take the form

57 $$ \frac{{d\phi_{2} \left( x \right)}}{d\,x} = \left[ {\begin{array}{*{20}c} { - \frac{{q_{0} }}{K}} & {{\text{for}}\,\,x = 0} \\ 0 & {{\text{for}}\,\,x = L} \\ \end{array} } \right] $$

The general solution of (56) takes the form

58 $$ \phi_{2} \left( x \right) = C_{1} \cosh \left( {\lambda x} \right) + C_{2} \sinh \left( {\lambda x} \right) + \frac{\gamma }{{\lambda^{2} }}\psi $$

Applying the boundary conditions (57) as follows:

59 $$ \left. {\frac{{d\phi_{2} }}{dx}} \right|_{x = 0} = \left. {C_{1} \lambda \sinh \left( {\lambda x} \right) + C_{2} \lambda \cosh \left( {\lambda x} \right)} \right|_{x = 0} = - \frac{{q_{0} }}{K} $$

and

60 $$ \left. {\frac{{d\phi_{2} }}{dx}} \right|_{x = L} = \left. {C_{1} \lambda \sinh \left( {\lambda x} \right) + C_{2} \lambda \cosh \left( {\lambda x} \right)} \right|_{x = L} = 0 $$

Hence, we have

61 $$ C_{2} = - \frac{{q_{0} }}{K\lambda } $$

and

62 $$ C_{1} = - C_{2} \frac{{\cosh \left( {\lambda L} \right)}}{{\sinh \left( {\lambda L} \right)}} = \frac{{q_{0} }}{K\lambda }\frac{{\cosh \left( {\lambda L} \right)}}{{\sinh \left( {\lambda L} \right)}} $$

Finally, we have

63 $$ \phi_{2} \left( x \right) = \frac{{q_{0} }}{K\lambda }\frac{{\cosh \left( {\lambda L} \right)}}{{\sinh \left( {\lambda L} \right)}}\cosh \left( {\lambda x} \right) - \frac{{q_{0} }}{K\lambda }\sinh \left( {\lambda x} \right) + \frac{\gamma }{{\lambda^{2} }}\psi $$

The last equation after simplification will take the form

64 $$ \phi_{2} \left( x \right) = \frac{{q_{0} \cosh \lambda \left( {L - x} \right)}}{K\lambda \sinh \lambda L} + \frac{\gamma }{{\lambda^{2} }}\psi $$
